# Blood Pressure Profile in School Children (6–16 Years) of Southern India: A Prospective Observational Study

**DOI:** 10.3389/fped.2015.00024

**Published:** 2015-03-31

**Authors:** Mohammad Sayeemuddin, Deepak Sharma, Aakash Pandita, Tabassum Sultana, Sweta Shastri

**Affiliations:** ^1^Candy Children Hospital, Hyderabad, Telangana, India; ^2^Department of Paediatrics, Pandit Bhagwat Dayal Sharma Post Graduate Institute of Medical Sciences, Haryana, India; ^3^Department of Paediatrics, Sri Maharaja Gulab Singh (SMGS) Hospital, Jammu, India; ^4^Mediciti Institute of Medical Sciences (MIMS), Hyderabad, India; ^5^ACPM Medical College, Dhule, Maharashtra, India

**Keywords:** blood pressure, nomogram, hypertension, systolic blood pressure, diastolic blood pressure, weight, height, BMI

## Abstract

**Aims and objective:** To determine normal blood pressure (BP) in apparently healthy, asymptomatic school children in the age group of 6–16 years and to determine the correlation of BP values with different sex, weight, height, and body mass index (BMI) and also to find out prevalence of hypertension in school going population.

**Materials and methods:** This prospective, observational study enrolled 3,302 urban children (1,658 boys and 1,644 girls) in the age group of 6–16 years. These were analyzed to study the distribution pattern of systolic blood pressure (SBP) and diastolic blood pressure (DBP) at different ages, sex, weight, height, and BMI. The SBP and DBP were noted as per age and sex. The association was seen between mean SBP and mean DBP with weight, height, and BMI. Information was collected about the family history of hypertension and was correlated with the obtained SBP and DBP readings.

**Results:** The mean SBP in males at 6 years was 99.69 ± 3.62 mm of Hg, at 10 years was 102.20 ± 2.16 mm of Hg, and at 16 years was 115.33 ± 1.26 mm of Hg. The mean SBP in females at 6 years was 96.55 ± 2.86 mm of Hg, at 10 years was 101.16 ± 2.12 mm of Hg, and at 16 years was 112.41 ± 1.06 mm of Hg. The correlation coefficient for relationship between age and SBP in males and females was 0.89 and 0.91, respectively, and for DBP was 0.92 and 0.90, respectively. The correlation coefficient for relationship between height and SBP in males and females was 0.91 and 0.93, respectively, and for DBP was 0.92 and 0.88, respectively. The correlation coefficient for relationship between weight and SBP in males and females was 0.92 and 0.92, respectively, and for DBP was 0.94 and 0.91, respectively. In the nomogram obtained in the study, 95% of study population fall between mean +2SD and −2SD.

**Conclusion:** The blood pressure (BP) (SBP and DBP) tends to increase with age, weight, height, and BMI. The BP values (SBP and DBP) increases grossly after 11 years of age. The students with positive family history of hypertension had higher valve when compared to other student. The BP of children and adolescents can be evaluated using the reference table according to age. The table provided helps to classify as “normal” or “hypertension” (>+2SD).

## Introduction

The measurement of blood pressure (BP) is firmly established as an important component of routine pediatric physical examination (1). BP is considerably lower in children than in adults but usually increases steadily throughout the first two decades of life (2–5). The BP is continuously distributed and BP profile in children varies with age, sex, weight, height, body mass index (BMI) (obesity), family history of hypertension, social economic status, and dietary habits. Local reference values have to be established to understand the BP variable (2, 6, 7). The prevalence of hypertension in children has been reported to be approximately 1–3%. Elevated BP in children and adolescents may be an early expression of essential hypertension in adulthood (2, 8, 9). Juvenile BP has been reported as among one of the several predictors of adult BP (10, 11). As it is not possible to record reliable BP by conventional methods in children below 6 years, hence the ideal age would be 6–16 years, i.e., school children. NIH of USA has recommended that BP measurement should be part with weight and height measurement, which is done in children at least once a year. But even today in many parts of world including India, this practice has not been implemented due to unknown reasons. Most of the studies have established the standards for children of western world but presently there are no such standards available for Indian children, and the western standards cannot be applied to Indian children, because of difference in factors such as ethnic, socioeconomic, dietetic, environmental, and emotional factors between Indian and Western countries. Hence, there is need to establish the normal BP standards for Indian children and to find out the prevalence of hypertension among them. Hence, the present study was taken up to determine normal BP in apparently healthy, asymptomatic school children in the age group of 6–16 years, and to determine the correlation of BP values with different sex, weight, height, and BMI and also to find out prevalence of hypertension in school going population.

## Materials and Methods

This was a community based prospective, observational study done in Department of Pediatrics, South Central Railway Hospital, Secunderabad, India. The study sample consisted of 3,302 (1,658 boys and 1,644 girls) apparently healthy school children, in the age group of 6–16 years, and the study was conducted from January 2010 to October 2010. The study was approved by the Institutional Ethical Committee and Institutional Research Board (IRB) and written consent was taken from child’s care taker or parents before enrollment. A short history about febrile illnesses, burning micturition, cough, and dyspnea/breathlessness was taken. A complete general physical examination from head to toe was done of all enrolled child. Vital parameter and temperature were recorded. Child was clinically assessed for anemia, jaundice, cyanosis, clubbing, lymphadenopathy, and edema and was looked for any congenital anomalies. A detailed systemic examination of all systems (respiratory system, cardiovascular system, gastro-intestinal system, nervous system) was done to exclude the systemic disorders like congenital heart disease, renal disorders, and liver diseases. The parameters that were studied included age, sex, weight, and height, BMI (kg/m^2^), systolic blood pressure (SBP), diastolic blood pressure (DBP). The inclusion criteria included healthy school children, in the age group of 6–16 years and the exclusion criteria included children <6 years and more than 16 years and failure to obtain consent from parents or care giver.

A written *pro forma* was sent home with the child to collect information about family history of hypertension (father/mother). Dietary history (vegetarian/non-vegetarian) and socioeconomic status (per capita income) and the performa were collected after 2 days. Age in completed years was recorded as per school admission registers. Measurements were made by a single person (who was trained prior for taking all measurement) and same equipment was used to obtain accurate measurement and to increase the sensitivity of the results. Weight was measured in kilograms using a dial type of weighing machine and all recordings including BP measurements was done by a single individual to eliminate observers subjective bias. Height was measured to nearest 1 cm with subject standing without shoes using a non-stretchable metallic tape.

### Measurement of blood pressure

Before recording BP, the procedure was explained to children and sufficient time was given to allay anxiety and fears. BP was measured in supine position using diamond mercury manometer with a set of different sized cuffs as per the recommendation given by the fourth report on the diagnosis, evaluation, and treatment of high BP in children and adolescents (2004) (2). The cuff bladder was wide enough to cover at least 2/3rd of arm and long enough to encircle arm completely. Auscultatory method was used and the first and fifth Korotkoff’s sounds were taken as indicative of the SBP and DBP, respectively. BP was recorded three times with 2 min interval between each measurement. In children, where a higher range of BP was observed, the factors like anxiety and fear were removed and rerecorded after 1 h. Average of three BP readings was taken, BMI was calculated using the formula [BMI = Weight (kg)/Height^2^ (m)].

#### Definition

There is no agreed definition of hypertension since BP is a continuous variable within a population (12, 13). Thus, hypertension is defined as “average systolic blood pressure and or diastolic blood pressure that is >95 percentile for gender, age, and height on ≥3 occasions” (2). Normal BP is defined as SBP and DBP that are <90 percentile for gender, age, and height (2).

### Statistical analysis

All the data were entered in Microsoft excel sheet and SPSS version 16 for window’s was used for analysis. Correlation coefficient and simple linear regression analysis was done for predicting BP (SBP and DBP) separately for age, sex, weight, height, and BMI. Norms were established for each individual age height, weight and BMI (kg/m^2^). The data were analyzed by Karl–Pearson’s coefficient of co-relation and regression.

## Results

In the present study, the prevalence of hypertension was found to be 2.42% (95th percentile for age and sex was cut-off point).

### Variation with age

In the study, 3,302 urban children [1,658 (50.2%) boys and 1,644(49.8%) girls] in the age group of 6–16 years were clinically examined and their BP was recorded. The correlation coefficient for relationship between age and SBP in males and females was 0.89 and 0.91, respectively, with significant *P*-value (*P* < 0.001). The mean DBP in males at 11 years is 65.03 ± 0.86 mm of Hg and at 16 years was 74 ± 1.08 mm of Hg (Table 1). The correlation coefficient for relationship between age and DBP in males and females was 0.92 and 0.90, respectively, with significant *P*-value (*P* < 0.001).

**Table 1:** **Table showing systolic blood pressure and diastolic blood pressure according to age and sex**.

Age (years)	Male			Female		
	No. of cases	Mean	SD	No. of cases	Mean	SD
**SYSTOLIC BLOOD PRESSURE**						
6	156	99.69	3.62	146	96.55	2.86
7	152	99.46	3.07	152	98.53	2.17
8	150	100.41	2.56	150	99.08	2.38
9	150	102.31	1.84	150	100.86	2.16
10	150	102.20	2.16	150	101.16	2.12
11	150	104.20	1.93	150	104.04	2.38
12	150	105.84	1.88	150	105.07	1.93
13	150	107.75	1.33	150	108.32	1.32
14	150	109.90	1.92	150	107.87	1.33
15	150	112.47	2.09	150	110.47	2.32
16	150	115.33	1.26	146	112.41	1.06
**DIASTOLIC BLOOD PRESSURE**						
6	156	60.11	3.64	146	57.97	2.62
7	152	61.13	1.61	152	59.46	1.39
8	150	62.37	1.45	150	61.18	1.69
9	150	63.30	1.11	150	61.59	1.08
10	150	64.59	1.00	150	62.56	1.13
11	150	65.03	0.86	150	63.34	1.55
12	150	66.18	0.87	150	64.01	1.64
13	150	68.12	0.86	150	65.01	1.63
14	150	69.27	0.86	150	65.24	1.35
15	150	71.86	1.09	150	66.07	1.20
16	150	74.00	1.08	146	70.11	1.54

### Variation with height

Based on height of the individual student, eight groups were made independent of age and weight with a difference of 10 cm between the groups. It was observed that there is not much increase in mean SBP up to 130 cm (both in males and females) and SBP increased significantly and gradually in children above 130 cm of height. The correlation coefficient for relationship between height and SBP in males and females was 0.91 and 0.93, respectively, with significant *P*-value (*P* < 0.001). The same findings were seen in the case of mean diastolic BP (Table 2). The correlation coefficient for relationship between height and DBP in males and females was 0.92 and 0.88, respectively, with significant *P*-value (*P* < 0.001).

**Table 2:** **Relation of mean systolic blood pressure and mean diastolic blood pressure to height**.

Height (cm)	Males			Females		
	No. of cases	Mean	SD	No. of cases	Mean	SD
**MEAN SYSTOLIC BLOOD PRESSURE**						
100–110	4	96.33	1.41	6	95.33	1.76
110–120	194	99.57	3.43	242	97.10	2.60
120–130	350	100.54	2.61	344	99.71	2.11
130–140	258	102.34	2.00	224	101.74	1.97
140–150	250	105.46	1.63	278	105.67	1.83
150–160	276	108.63	1.69	504	109.69	2.20
160–170	308	113.61	2.13	46	112.87	2.18
170–180	18	116.37	1.16	–	–	–
**MEAN DIASTOLIC BLOOD PRESSURE**						
100–110	4	56.67	1.89	6	57.78	1.69
110–120	194	60.39	3.28	242	58.41	2.19
120–130	350	62.64	1.45	344	61.21	1.47
130–140	258	64.64	1.08	224	62.84	1.34
140–150	250	65.91	0.98	278	63.88	1.62
150–160	276	68.47	1.20	504	67.47	2.19
160–170	308	72.56	1.76	46	70.29	1.69
170–180	18	73.85	1.19	–	–	–

### Variation with weight

The weight of students was divided into nine groups, independent of age and height of the children with a difference of 5 kg between each group. The mean SBP and DBP were calculated. In this study, it was observed that the mean SBP and mean DBP in both males and females increased gradually from 15 to 60 kg weight (Table 3). The correlation coefficient for relationship between weight and SBP in males and females is 0.92 and 0.92, respectively, with a significant *P*-value (*P* < 0.001). The correlation coefficient for relationship between weight and DBP in males and females was 0.94 and 0.91, respectively, with a significant *P*-value (*P* < 0.001).

**Table 3:** **Relation of mean systolic blood pressure and mean diastolic blood pressure to weight**.

Weight (kg)	Males			Females		
	No. of cases	Mean	SD	No. of cases	Mean	SD
**MEAN SYSTOLIC BLOOD PRESSURE**						
15–20	112	98.33	2.87	178	96.85	2.59
20–25	428	100.53	2.80	424	99.49	2.46
25–30	226	102.48	2.07	174	101.73	2.33
30–35	204	104.69	1.97	194	104.21	2.06
35–40	154	106.76	1.51	156	106.92	1.95
40–45	188	108.76	1.73	254	108.31	1.50
45–50	146	112.04	1.97	248	111.56	1.94
50–55	188	114.62	1.97	16	113.17	1.11
55–60	12	115.00	1.10	–	–	–
**MEAN DIASTOLIC BLOOD PRESSURE**						
15–20	112	59.31	2.95	178	58.03	1.79
20–25	428	62.34	1.95	424	60.88	1.84
25–30	226	64.25	0.91	174	62.74	1.16
30–35	204	65.37	0.81	194	63.24	1.50
35–40	154	67.00	0.75	156	65.06	1.29
40–45	188	68.79	0.67	254	66.01	1.47
45–50	146	70.95	1.13	248	69.38	1.31
50–55	188	73.60	1.04	16	72.25	0.79
55–60	12	75.33	0.60	–	–	–

### Variation with BMI

The BMI of students was divided into five groups, with a difference of 2 kg/m^2^ between each group. The mean SBP and DBP were calculated. It was observed that as BMI increased, both SBP and DBP increased gradually and significantly (Table 4). The correlation coefficient for relationship between BMI and SBP in males and females is 0.83 and 0.82, respectively, with significant *P*-value (*P* < 0.001). The correlation coefficient for relationship between BMI and DBP in males and females was 0.89 and 0.85, respectively, with significant *P*-value (*P* < 0.001).

**Table 4:** **Relation of mean systolic blood pressure and mean diastolic blood pressure to BMI**.

BMI	Males			Females		
	No. of cases	Mean	SD	No. of cases	Mean	SD
**MEAN SYSTOLIC BLOOD PRESSURE**						
12–14	90	98.56	2.48	182	98.59	3.06
14–16	750	101.71	3.23	682	100.58	3.36
16–18	468	107.27	3.64	408	106.34	3.49
18–20	324	112.49	3.35	346	110.45	2.50
20–21	26	112.56	2.43	26	110.46	3.46
**MEAN DIASTOLIC BLOOD PRESSURE**						
12–14	90	60.56	2.45	182	59.38	1.71
14–16	750	63.04	2.43	682	61.42	2.39
16–18	468	67.42	2.11	408	64.80	2.13
18–20	324	71.98	2.20	346	68.32	2.22
20–21	26	74.05	1.35	26	70.26	1.60

**Figure 1:**
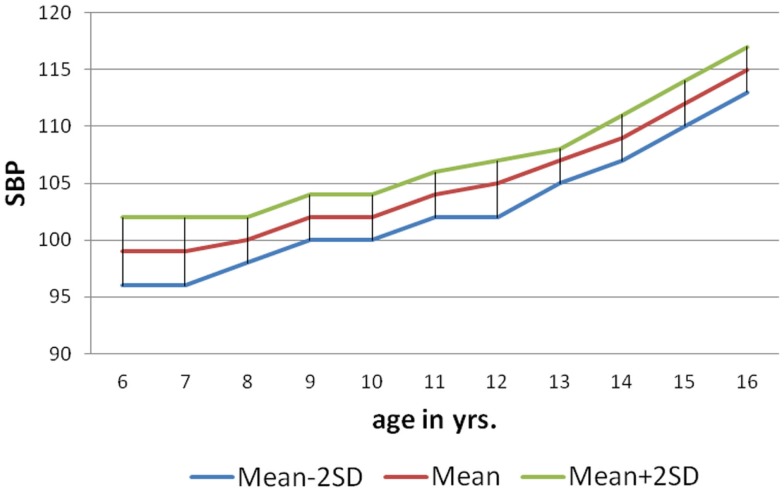
**Norms of mean SBP according to age in boys (6–16 years)**.

**Figure 2:**
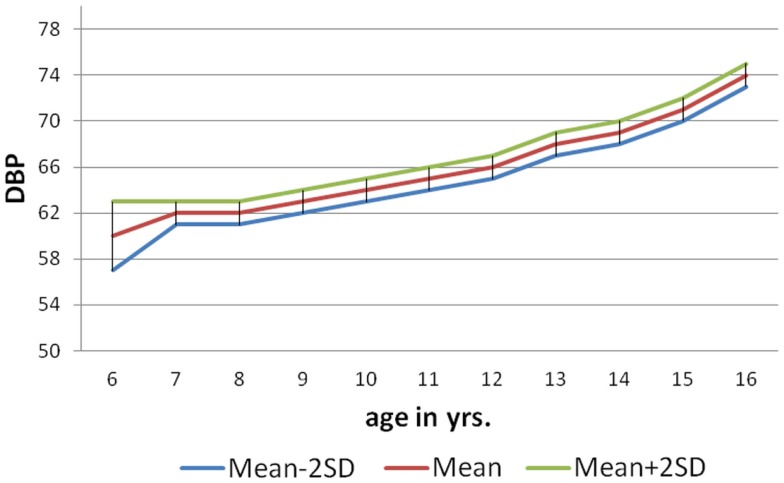
**Norms of mean DBP according to age in boys (6–16 years)**.

**Figure 3:**
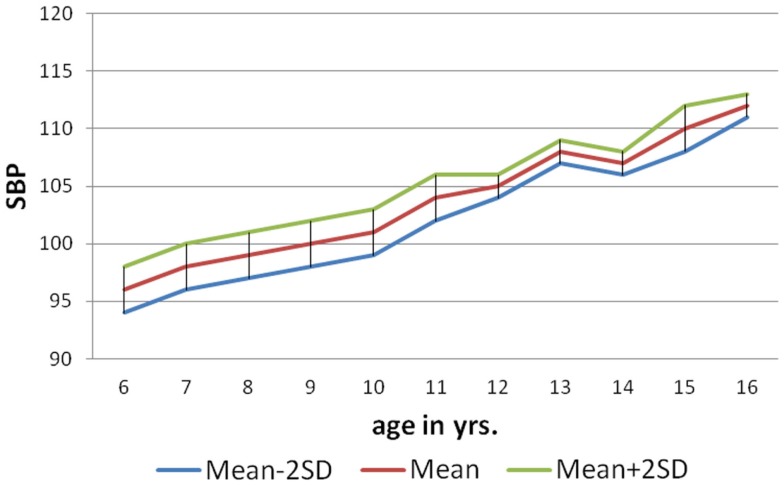
**Norms of mean SBP according to age in girls (6–16 years)**.

**Figure 4:**
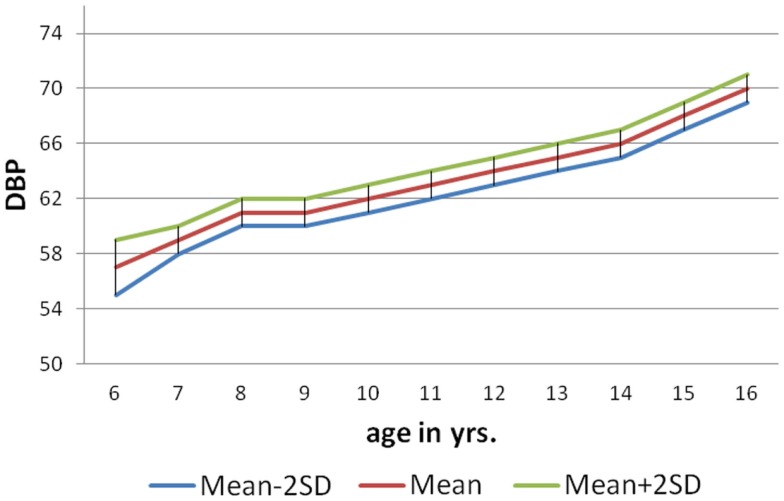
**Norms of mean DBP according to age in girls (6–16 years)**.

As per the information received through the *pro forma* sent home with each child to collect about family history of hypertension (father/mother), 146 (9%) of male and 84 (5%) females students had positive family history of hypertension. The mean SBP of males and females with positive family history of hypertension at 6 years was 104.4 ± 1.04 and 100.7 ± 1.26 mm of Hg, respectively, and for that of 10 years 116.8 ± 0.85 mm of Hg (males) and 113.3 ± 1.13 mm of Hg (females). The mean DBP of males and females with positive family history of hypertension at 6 years was 64.50 ± 0.98 and 62.19 ± 1.34 mm of Hg, respectively, and for that of 16 years was 73.56 ± 1.13 mm of Hg (males) and 71 ± 1.42 mm of Hg (females). The nomogram charts were made for both male and female as per age with mean SBP ± 2SD and mean DBP ± 2SD (Figures 1–4). The 95% of the study population falls between these two limits.

## Discussion

In the present study, the data concerning the readings of BP of children at different age group are presented. The SBP and DBP measurements in our study according to age and sex, obtained by auscultation using the sphygmomanometer are comparable to studies done by Sharma et al. (5), Krishna et al. (8), Gupta et al. (14), and Rosner et al. (15).

In the present study, both SBP and DBP show a positive correlation with increase in age, consistent with the findings reported by several studies (5, 16–18). Male students had 1–2 mm of Hg of higher BP when compared with their female counterparts at all ages. This is similar to the average annual increase of 2 mm of Hg in boys and about 1 mm of Hg in girls reported by World Health Organization study group (18). Thus, it can be concluded that the males had slightly higher values of BP (1–2 mm of Hg) when compared to the females for that age. This could be explained because boys are heavier and taller when compared to females for that age which results in this observation. It was also observed from the results of mean SBP for different ages (in either sex) that there is not much increase (1–2 mm of Hg/year) in the SBP between age groups 6–10 years. But the rise is steeper (2–3 mm of Hg/year) during adolescence (>11 years) when compared with the rise of SBP between age group of 6–10 years. In the present study, there was a minimal increase of 4–5 mm of Hg in diastolic BP from age 6 to 10 years in both sexes. But diastolic BP showed a spurt from 11 years (adolescence) and it increased for 8–10 mm of Hg, which correlated with results obtained by Sharma et al. (5) and Krishna et al (8). The spurt in SBP and DBP may be possibly due to age related hormonal, physical, and psychological changes occurring in the body during puberty (>11 years). For DBP, a rising trend with age was also present, although this rise was much less marked than the SBP and this similar finding was reported in other studies (5, 18). Height is related to BP and is an independent variable for BP (2, 17). In our study, the mean SBP and DBP in both sexes increased 3–5 mm of Hg for every 10 cm increase in height, independent of age, and weight. It was also observed that mean SBP and DBP showed an increase of 3–4 mm of Hg up to an height of 130 cm in both sex and BP increment was more pronounced (4–5 mm of Hg) in students whose height was more than 130 cm (in either of sex). This probably could be explained as BP does not have a simple linear correlation with height as it is thought to be or other factors like hormonal, emotional factors can be credited for this observation. In our study, the relation of SBP and DBP with height was independent of age. Similar results were shown by various studies conducted till now (5, 8, 17, 19). Hence height has to be considered independent of age before classifying the child as pre-hypertensive/hypertensive, which means taller children are allowed higher normal BP when their height is taken into consideration than when age is used alone. On the other hand, shorter children and adolescents are identified as having high normal or mildly elevated BP when only age and sex derived data is used. Thus, the BP nomogram obtained according to height should is always recommended to be used in pediatric practice.

In our study, there was an increase in 2.5–3 mm of Hg in DBP an every weight group up to 60 kg. The study done by Agarwal et al. (17) also showed a similar trend, but the increase in mean SBP and DBP was 1–1.5 mm of Hg and 1.5–2 mm of Hg, respectively, with increase of every 5 kg weight. The difference was possibly due to the present study was done in an urban private school where all the students belong to higher socioeconomic status (Grade I modified Prasad classification). It is known that higher BP values are described in families belonging to higher socioeconomic status due to nutritional and psychological factors. The other reason could be that Agarwal et al. (17) used Korotkoff’s phase IV to determine DBP, but we used Korotkoff’s phase V as DBP. In our study, it was also noted that the increase in DBP according to weight was more pronounced than increase in SBP (2 versus 3 mm of Hg), which was unexplainable. Heart size is closely associated with the body size (2, 20). The body size is objectively measured by BMI, which is a measure of obesity. It is well known that obesity is associated with stroke, atherosclerosis, coronary artery disease, and endocrinal disorders. The association between hypertension and obesity in adults is well established. But relationship between hypertension and obesity in childhood has been noted, but less extensively evaluated (2, 21, 22). In our study, the mean SBP and DBP increased by 3–4 mm of Hg with increase in every 2 kg/m^2^ of BMI (each group). This study shows a positive linear association of BMI with SBP and DBP in both sexes and these results were comparable to other studies (5, 8, 14, 19). Thus, concluding that obese children have higher levels of BP when compared to their normal counterparts. However, there is paucity of information about the possible mechanism to relate obesity and higher BP values. The postulation, which have been given, includes increased cardiac output, increased blood volume, excessive sodium intake as a consequence of excessive calorie intake, increased steroid production, and alteration in receptors in various pressure substances but these hypothesis still requires firm scientific basis to validate them (14, 21, 22).

In our observation, the mean values of SBP and DBP for age in males and females with positive family history of hypertension is 2–3 mm of Hg higher for each group when compared to the mean values of SBP and DBP of general study population (from 6 to 16 years). Thus, the BP values are at higher range in both sexes when compared to general population. Similar results were shown by studies done by others (14, 18). Thus, proving that the familial tendency of elevated BP can be detected early in life (first and second decade). This could be attributed to the nutritional habits, life style of the family members, and genetic predisposition. Thus, it is recommended to modify the life style and dietary habits of children with positive family history of hypertension to prevent the disastrous complications of hypertension during adulthood. Based on the predictive equation, norms were obtained for both SBP and DBP based on the observed readings and the upper and lower limits of SBP and DBP for that age was obtained for the local population from 6 to 16 years age group.

The limitation of the study includes as the study population was from higher socioeconomic class, hence we cannot extrapolate the results to lower and middle income group children. There was a lack of follow up of the children’s who had hypertension. We did not perform multi-regression analysis on the data; hence there could have been some unseen confounders in the study.

## Conclusion

Blood pressure in an important vital sign, which reflects the integrity of the cardiovascular system, renal, endocrinal system, and other systems in the body. After critically analyzing the results of the present study and comparing the results with other studies, it can be concluded that BP, both systolic and diastolic gradually increases with age, the increase being more pronounced in SBP than in DBP. The increase in the BP with increase in the age is not uniform with a wide range of fluctuations, between different age groups and with a spurt in SBP with the onset of puberty. There is no significant difference in BP of the two sexes when the values are corrected for maturation status. There is a strong correlation between BP and weight and BP and height in both sexes (*R* > 0.7 and *P* < 0.001). When BP values are arranged according to weight and height criteria, the correlation with age disappears. Hence body weight and height are the principle determinants of BP.

## Author Contributions

MS wrote the first draft of the manuscript. DS and AP helped in writing manuscript and did primary corrections in the manuscript. TS and SS made final corrections of manuscript before submission. All the authors approved the submission of this version of the manuscript and takes full responsibility for the manuscript. There was no external funding, honorarium, grant, or other form of payment given to anyone to produce the manuscript.

## Conflict of Interest Statement

There are no prior publications or submissions with any overlapping information, including studies and patients. The manuscript has not been and will not be submitted to any other journal while it is under consideration by Frontiers in Pediatrics. The authors declare that the research was conducted in the absence of any commercial or financial relationships that could be construed as a potential conflict of interest.
